# Effect of medical staff training on vaccination coverage in outpatients with cancer: An interventional multicenter before-and-after study

**DOI:** 10.1016/j.jvacx.2023.100261

**Published:** 2023-01-08

**Authors:** Pierre Rivière, Nicolas Penel, Karine Faure, Guillaume Marie, Abeer Najem, Marie-Karelle Rivière, Sophie Panaget

**Affiliations:** a Medical Oncology Department, Boulogne-sur-Mer Hospital, Boulogne-sur-Mer, France; b Lille University (Medical School) and Medical Oncology Department, Centre Oscar Lambret, France; c Infectious Diseases Department, Lille University Hospital, Lille, France; d Saryga, Paris, France

**Keywords:** Vaccination coverage, Influenza, *Streptococcus pneumoniae*, Cancer, Chemotherapy, Medical training, DTP, Diphtheria-Tetanus-Pertussis, PS, performance status, VC, vaccine coverage, WHO, World Health Organization

## Abstract

**Purpose:**

Despite widely disseminated guidelines, pneumococcal and influenza vaccination coverage (VC) remains insufficient in patients with cancer receiving cancer treatment. We performed an interventional study to evaluate VC in patients with cancer treated at the medical oncology departments of three North-of-France hospitals and to assess the effect of medical staff training on VC in these patients.

**Methods:**

A standardized questionnaire assessed VC in adult patients with cancer receiving anticancer treatment at three day hospitals during December 2–7, 2019. Subsequently (January 2020), we organized educational training sessions for medical staff from each hospital to discuss the current vaccination guidelines. To assess the impact of training on pneumococcal and influenza VC, we re-administered the same questionnaire in March 2020. Because there are no specific guidelines on Diphtheria-Tetanus-Pertussis (DTP) vaccination and no improvement was expected, DTP VC acted as an internal control.

**Results:**

In total, 272 patients from all three hospitals were enrolled in the “before study”; 156 patients from only two hospitals were enrolled in the “after study” as medical training and data collection at the third were impossible because of administrative reasons and COVID-19 pandemic. The predictors were age for DTP VC; treatment center for pneumococcal VC; and age, sex, and tumor histology (adenocarcinoma vs. others) for influenza VC. Neither influenza VC (42.6% vs. 55.1%, p = 0.08), nor pneumococcal VC were significantly improved post-intervention (11.8% vs. 15.4%, p = 1). There seems to be a small effect in the most fragile for influenza VC.

**Conclusion:**

As expected, VC was very low in patients with cancer, consistent with the literature. There was no impact of the intervention for pneumococcal and influenza VC.

## Introduction

In addition to the cancer itself, chemotherapy causes a variable degree of immunosuppression, depending on age, tumor pathology, and the type of chemotherapy, resulting in increased risks of infection, morbidity, and mortality [1].

Influenza and pneumococcal vaccinations in patients with cancer are critical given the risk of developing life-threatening infections causing prolonged hospital stay and anticancer treatment delay. Vaccination recommendations for patients undergoing chemotherapy, include the vaccines recommended for the general population, influenza and pneumococcal vaccinations. Live viral vaccines should not be administered in patients receiving chemotherapy. Three months after cancer chemotherapy, patients should be re-vaccinated with inactivated vaccines and the live vaccines for varicella, measles, mumps, and rubella according to the annual schedule that is routinely indicated for immunocompetent persons [2], [3]. Guidelines in France also recommend a second vaccine dose for preventing influenza during the peak of the influenza season. Despite these mitigation efforts, 15%–20% of patients with influenza require hospitalization [4]. In addition to higher hospitalization rates, immunocompromised individuals may experience mortality rates of up to 50% and delays in chemotherapy schedules. A *meta*-analysis showed a 70% decrease in the incidence of influenza-like illnesses in vaccinated individuals compared with non-vaccinated individuals [5]. A retrospective study of 1,225 patients with colorectal cancer who underwent chemotherapy found a lower incidence of pneumonia, lower mortality at 1 year, and fewer treatment interruptions in vaccinated than in unvaccinated patients [6].

Despite widely disseminated guidelines, pneumococcal and influenza vaccination coverage (VC) remains insufficient in patients with cancer receiving chemotherapy. Several studies have addressed VC issues in patients with cancer, particularly for influenza and pneumococcal infection, all of which demonstrated insufficient VC in patients undergoing chemotherapy [6], [7], [8], [9], [10], [11], [12], [13], [14], [15], [16]. VC against influenza is approximately 30%, whereas that against pneumococcus varies between 5% and 15%. For pneumococcal vaccination, the relative risk of invasive pneumococcal infection in a patient receiving chemotherapy for solid cancer is up to 23 [17]. Even with insufficient VC against pneumococcus, Sangil et al. showed a decrease in the incidence of invasive pneumococcal infections from 20/100,000 to 8/100,000 inhabitants [7].

Improving VC in patients with cancer treated with chemotherapy is important for reducing morbidity and mortality; ensuring proper training of medical staff is critical in this setting. A study of general clinical practice in the Netherlands found that a large proportion (48%) of general practitioners felt that the responsibility of vaccinating patients against influenza lay with the treating oncologist [9]. Furthermore, physicians are requesting additional professional training to improve their knowledge about vaccination [8], [10], [11]. Thus, we conducted an interventional, multicenter, before-and-after study to evaluate pneumococcal and influenza VC in patients with cancer treated with chemotherapy, hormone therapy, target therapy or immunotherapy and to assess the effect of medical staff training on VC in these patients. As there is no recommendation for Diphtheria-Tetanus-Pertussis (DTP) vaccination, it served as an internal control.

## Material and methods

We conducted an interventional before-and-after study at the medical oncology departments of three North-of-France hospitals. Evaluations occurred before and after providing training to physicians to assess and improve VC in cancer outpatients. The three hospitals were Boulogne-sur-Mer Tertiary Hospital, Lille University Hospital, and Lille Comprehensive Cancer Center (Centre Oscar Lambret).

The first VC assessment occurred over a 1-week period during December 2–9, 2019. Between the first and second VC assessments, in January 2020, we organized training sessions with physicians to discuss the current vaccination guidelines. We also provided a vaccination protocol validated by our team of infectious disease specialists.

The second VC assessment occurred in March 2020, 8 weeks after the training sessions and 2 weeks before lock-down in France. Unfortunately, for administrative reasons, the training of the third center was not possible at the same time than the other two centers and was planned one month later. Due to the implementation of national containment protocols related to the COVID-19 pandemic, the training was not feasible and the third center could not be re-evaluated on time. Therefore, it was decided to exclude this center from the comparative analysis to avoid bias.

The same questionnaire, used during each of the two evaluation weeks, assessed the following characteristics: age, sex, and World Health Organization (WHO) Performance Status (PS) of the patient, histological type, stage (localized or metastatic), and primary site of the cancer and any ongoing cancer treatment (chemotherapy, hormone therapy, targeted therapy, and immunotherapy). Regarding the evaluation of vaccinations, the questionnaire assessed whether each patient’s vaccinations were up-to-date against DTP, seasonal influenza, and pneumococcus. One question that inquired whether the patient’s relatives had been contacted to update their vaccinations was misunderstood by some of the participants; therefore, it was excluded from the statistical analyses.

### Inclusion/exclusion criteria

The inclusion criterion was as follows: any patient over 18 years old undergoing oncological treatment presenting to the day hospital unit during the evaluation week. The exclusion criteria were as follows: any patient who was a minor or who refused to participate in the study and a lack of oncological treatment.

The main objective was to assess the influenza and pneumococcal VC of patients undergoing anticancer treatment at the three centers. The secondary objectives were to reassess the VC after the physicians underwent the training sessions, assess the impact of this training, determine the factors related to VC, and identify means of improving practices.

As there were no specific guidelines on DTP vaccination and we did not expect an improvement, DTP VC was used as an internal control.

### Ethics

The present study was approved by the Clinical Research and Innovation Department of each treatment center. In accordance with French regulations, this study was also approved by the Ethics Committee (Commission Nationale de l'Informatique et des Libertés). Informed consent was obtained from all participants. The authors certify that the study was performed in accordance with the ethical standards as laid down in the 1964 Declaration of Helsinki and its later amendments or comparable ethical standards.

An upstream estimation of the number of patients presenting at the day hospitals of each treatment center determined that over 1 week, 500 patients presented at different centers. We expected the inclusion of 50% of patients per week of assessment.

The completed questionnaires were collected from each center at the end of each evaluation week. Each patient was anonymized to integrate their information into a database and allow for statistical analysis.

### Statistical analysis

Detailed descriptive statistics of the population characteristics were calculated before and after the study. For the determination of VC predictors, data from the before and after study periods were merged. To restrict the number of possible covariates in the multivariate regression models, a pre-selection of potential VC predictors (p < 0.20) was performed using univariate logistic regressions for influenza, pneumococcal, and DTP factors. Next, multivariate logistic regression analyses were conducted using a stepwise procedure to identify sets of predictors of VC (p < 0.05) for influenza, pneumococcal infection, and DTP. Finally, the impact of the intervention (physician training) was evaluated as follows: (a) a test comparing two proportions (before/after) using Pearson's chi-squared test statistic and (b) logistic regression explaining the vaccination status as a function of the study period (before/after); both approaches were expected to generate concordant results. As one center could not be re-evaluated, it was removed from this analysis. Interaction terms were considered in the multivariate logistic regression model to explain possible differences in vaccination before and after the intervention.

## Results

**Fig. 1:**
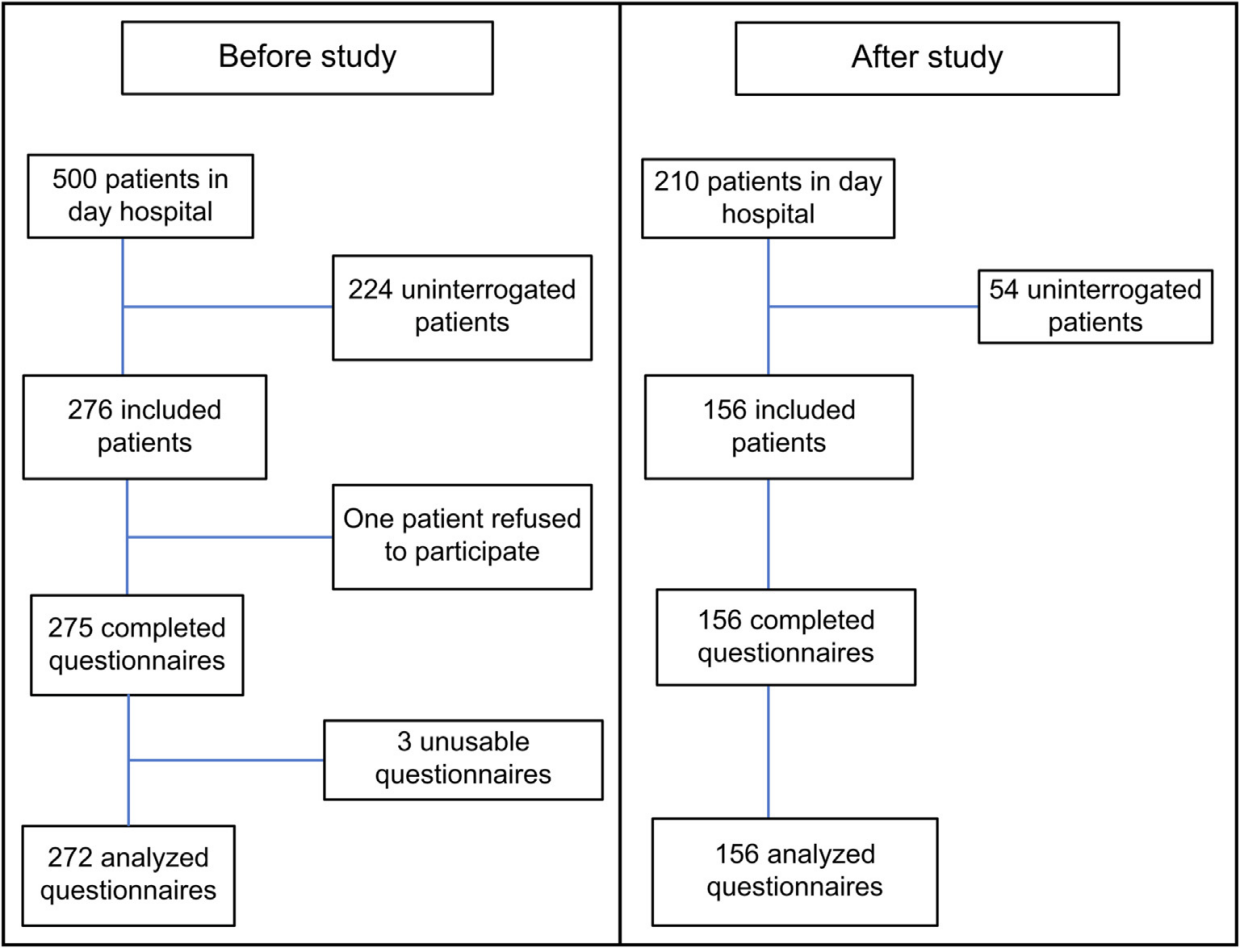
Flow chart of the number of patients included in the before and after studies to assess the impact of medical staff training on vaccine coverage.

For the “before” period, out of 500 patients presenting at the day hospital, 276 were asked to complete the questionnaire. One patient refused to participate; thus, 275 questionnaires were collected. Of these, 272 were usable. Three questionnaires were excluded from the analyses because they were incomplete. For the “after” period, out of 210 patients presenting at the day hospital, data from 156 patients were included; none refused to participate, and all questionnaires were usable. These data are presented in a flow chart (Fig. 1).

The characteristics of the patients included in the “before” and “after” periods of the study are listed in Table 1. In the first evaluation, 272 patients were included, whereas 156 were included in the second. This discrepancy can be explained by the fact that the reassessment could not be completed at one of the centers. The population characteristics were homogeneous for the before and after periods. The median age was 65 years for both evaluations. Equal proportions of male and female patients were included. The repartition of the PS was similar for both evaluations. The primary site of the lesions was more often in the digestive tract than in the head and neck, breast, or gynecological area. The most common histological types were adenocarcinomas, followed by squamous cell carcinomas and other types. In both evaluations, three-quarters of patients had metastatic cancer. Patients more often received chemotherapy than combined chemo- and targeted therapy, targeted therapy alone, or immunotherapy. The data revealed that even though one of the centers could not participate in the second evaluation, the included patients appeared to be comparable in terms of their characteristics.

**Table 1:** Characteristics of the patients included in the before-and-after studies.

	**Before study** **(N = 272)**	**After study** **(N = 156)**	Baseline comparison* (p-value)
Age (years)			0.71
Mean (standard deviation)	63.4 (11.8)	63.9 (11.3)	
Median	65.0	65.0	
Minimum; Maximum	21; 91	36; 87	
Age by age group (years) [n (%)]			0.95
<65	132 (48.5)	77 (49.4)	
≥65	140 (51.5)	79 (50.6)	
Sex [n (%)]			0.59
Female	143 (52.6)	77 (49.4)	
Male	129 (47.4)	79 (50.6)	
World Health Organization Performance Status [n (%)]			0.04
0	103 (37.9)	40 (25.6)	
1	141 (51.8)	97 (62.2)	
2	28 (10.3)	19 (12.2)	
Primary site [n (%)]			0.17
CUP	1 (0.4)	1 (0.6)	
Brain	0 (0.0)	1 (0.6)	
Digestive tract	134 (49.3)	83 (53.2)	
Gynecological area	36 (13.2)	16 (10.3)	
Head and neck	38 (14.0)	22 (14.1)	
Bone	1 (0.4)	0 (0.0)	
Skin	0 (0.0)	1 (0.6)	
Pleura	1 (0.4)	0 (0.0)	
Lung	13 (4.8)	0 (0.0)	
Breast	35 (12.9)	25 (16.0)	
Soft tissue	2 (0.7)	0 (0.0)	
Urologic system	11 (4.0)	7 (4.5)	
Histological type [n (%)]			0.19
Adenocarcinoma	180 (66.2)	104 (66.6)	
Squamous cell carcinoma	54 (19.9)	26 (16.7)	
Others	38 (13.9)	26 (16,7)	
Stage of the disease [n (%)]			0.42
Localized	81 (29.8)	40 (25.6)	
Metastatic	191 (70.2)	116 (74.4)	
Treatment [n (%)]			0.23
Chemotherapy	172 (63.2)	103 (66.0)	
Chemotherapy + immunotherapy	0 (0.0)	1 (0.7)	
Chemotherapy + radiotherapy	3 (1.1)	0 (0.0)	
Chemotherapy + targeted therapy	47 (17.3)	25 (16.0)	
Hormone therapy + targeted therapy	1 (0.4)	0 (0.0)	
Immunotherapy	20 (7.4)	5 (3.2)	
Targeted therapy	29 (10.7)	22 (14.1)	
Center [n (%)]			*< 10^-16^*
Tertiary hospital	94 (34.6)	75 (48.1)	
University hospital	67 (24.6)	81 (51.9)	
Comprehensive cancer center	111 (40.8)	0 (0.0)	

Abbreviation: CUP, carcinoma of unknown primary.

* Pvalues associated to baseline characteristics comparison between “before study” and “after study” are non-significant at a 2.5% level, except for Center which is expected as one center could not be re-evaluated in “after study” due to Covid-19 pandemic.

The VC results for both time points are presented in Table 2. Before training, the DTP VC was 37.1%, influenza VC was 42.6%, and all patients received an injection in the fall. None of the patients received two injections. The pneumococcal VC was 11.8% (40.6% received an injection of 13-valent conjugate vaccine alone, whereas 59.4% received the full regimen).

**Table 2:** Vaccination coverage in the before and after studies.

	**Before study (N = 272)**	**After study (N = 156)**	**Uncorrected** **p-values (before vs after) with χ^2^ test**
DTP VC [n (%)]			
Yes	101 (37.1)	60 (38.5)	
No	171 (62.9)	96 (61.5)	
Influenza VC [n (%)]			0.08
Yes	116 (42.6)	86 (55.1)	
In the fall	116 (100.0)	79 (91.9)	
At least one injection	116 (100.0)	86 (100.0)	
Revaccinated if in endemic period	0 (0.0)	0 (0.0)	
No	156 (57.4)	70 (44.9)	
Pneumococcal VC [n (%)]			1
Yes	32 (11.8)	24 (15.4)	
13-valent conjugate only	13 (40.6)	4 (16.7)	
Full regimen completed	19 (59.4)	20 (83.3)	
No	240 (88.2)	132 (84.6)	

Abbreviations: DTP, Diphtheria-Tetanus-Pertussis; VC, vaccination coverage.

After training, the DTP VC was 38.5% and the influenza VC was 55.1%. A total of 91.9% of patients received an injection in the fall, whereas 8.1% of patients received the injection in the winter; none received two injections. The pneumococcal VC was 15.4% (16.7% received an injection of 13-valent conjugate vaccine alone, whereas 83.3% received the complete regimen).

For influenza VC, age (p < 0.0001), sex (p = 0.0036), and histologic type (p = 0.0128) were identified as predictors by the multivariate logistic regression analyses. As expected, older patients, as they are more fragile, were vaccinated at significantly higher rates for influenza. In addition, these patients satisfy two of the criteria for which vaccination is recommended in an organized global campaign, including receiving cancer treatment and being older than 65 years old. Men were vaccinated at significantly higher rates for influenza than women, which may be owing to the presence of other comorbidities, such as diabetes, obesity, and organ failure, that were not considered in our study. We also noted that the histologic type of cancer was a significant predictor (adenocarcinoma vs. others).

For pneumococcal VC, we only observed an effect related to the treatment center (p < 0.0001). Patients from Boulogne-sur-Mer hospital tended to be vaccinated at higher rates than those from the other two centers.

Age was identified as a predictor of DTP VC. There is no specific recommendation for DTP vaccination in patients undergoing anticancer treatment. These patients, identified as being more fragile, may have been vaccinated by their general practitioner.

The focus of the evaluation of the impact of the intervention (physicians’ training) was on influenza and pneumococcus VC. A test comparing the two proportions of vaccinated patients before and after the intervention was performed. No significant effect of the intervention was observed for influenza (p = 0.08) nor for pneumococcal infection (p = 1). As we performed two tests, corrected p-values could be used to account for multiplicity, but with the same conclusions. Thus, the intervention did not have an impact on influenza nor pneumococcal VC.

We also constructed a logistic regression model to explain the vaccination status as a function of the study period (before or after) to determine the impact of the intervention. The results were similar, since influenza VC (p = 0.06) and pneumococcal VC were not modified (p = 0.91).

**Fig. 2:**
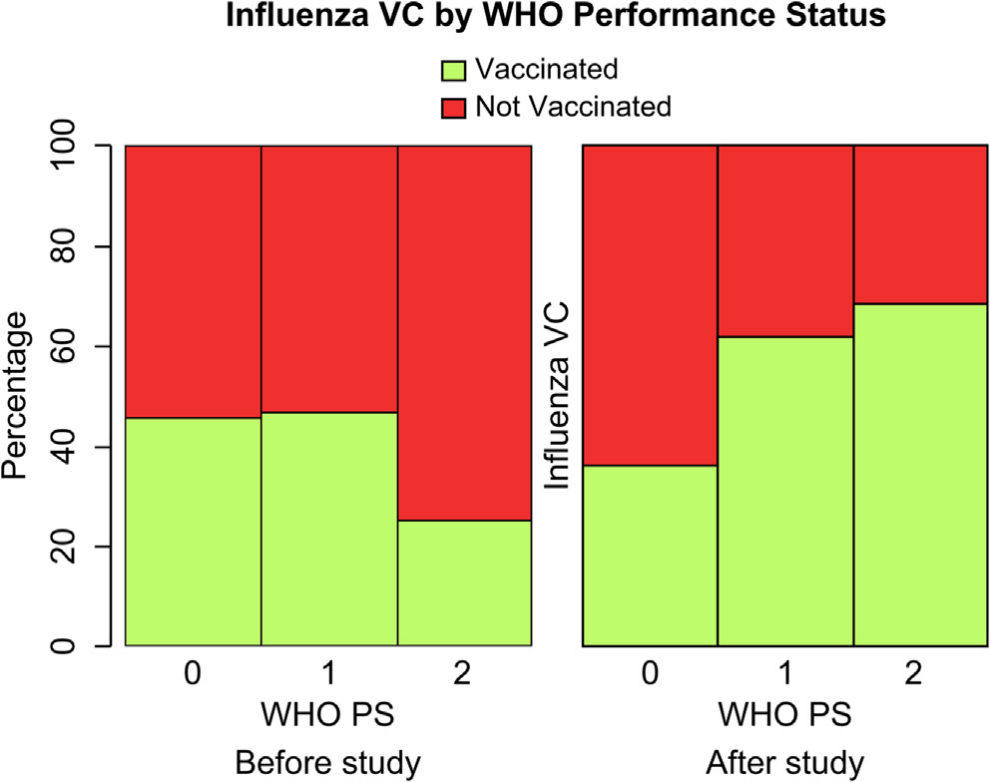
Influenza VC by WHO PS and study period. Abbreviations: VC, vaccination coverage; WHO PS, World Health Organization Performance Status.

By including interaction terms in the logistic model, patients with a higher WHO PS showed significantly higher vaccination rates than patients with a lower PS after the intervention (p = 0.009). Oncologists might recommend influenza vaccination more strongly to patients who are in poor condition (Fig. 2).

## Discussion

Our study confirms that VC is low in patients with solid tumors. Before training, DTP, influenza, and pneumococcal VC was 37.1%, 42.1%, and 11.8%, respectively, whereas after training, it was 38.5%, 55.1%, and 15.4%, respectively. No patient received two injections of the vaccine against influenza. The predictor for DTP VC was age; for influenza VC, the predictors were age, sex, and histological type. Our analysis highlighted a significant improvement in VC after the intervention, especially in patients with poor PS, but only for influenza, not for pneumococcal infection.

Our results are consistent with those in the literature [7], [8], [9], [10], [18], [19]. Despite the improvement, the VC before and after the intervention remained low. Regarding DTP, the VC in this study was comparable to that in other cancer studies and the general population. For example, a recent study by Monier et al. [13] found a DTP VC of 33.1% in oncology patients. In comparison, in a survey conducted by the Sanitary Surveillance Institute (Institut National de Veille Sanitaire) in January 2011, 44% of patients over 65 years of age were vaccinated against DTP in the general population [20].

In our study, the VC of influenza was higher than that reported by most investigations in medical oncology patients [6], [8], [9], [10], [13], [14], [15]. However, this difference cannot be explained by age because, in the other studies, half of the participants were also over 65 years old and, therefore, had another indication for receiving influenza vaccinations. In a study by Alkan et al., factors associated with low VC against influenza were age below 65 years, insufficiently informed oncologist, and doubts about the effectiveness of the vaccine among medical staff [18]. However, in a study conducted by Toleman et al. on patients with cancer in the UK, influenza VC was 68.1% [19]. In France, a free influenza vaccination campaign is conducted yearly from early October to late February. Eligible patients targeted for vaccination are those at risk of complications: pregnant women; patients aged 65 years and older; patients with chronic diseases; immunocompromised patients and their relatives; patients with obesity; patients living in a healthcare institution, group, or cruise ship travelers; and healthcare professionals. During the 2019–2020 influenza vaccination campaign, the VC for high-risk patients was 47.80% (31% before age 65 and 52% after age 65) [21].

In our study, pneumococcal VC was comparable to that in some other studies, i.e., approximately 5%–15% in patients with cancer [10], [13], [16]. However, other studies have found a higher pneumococcal VC. For example, Toleman et al. reported a pneumococcal VC of approximately 25% in those receiving treatment for cancer [19]. In another study that evaluated the VC of 429 patients without cancer at high risk of infections (i.e., those with diabetes, HIV, transplantation, heart failure, chronic kidney disease, solid organ transplantation, and chronic obstructive pulmonary disease), the pneumococcal VC was 32%, which is higher than the value in the present study [22].

Overall, our study found that medical staff training did not improve VC in patients with cancer. Toleman et al. also conducted a before-and-after study of VC after the dissemination of recommendations for vaccination [19]. They found that influenza VC increased from 71.6% at the first reassessment (January 2013) to 72.7% at the second (April 2014), a change that was not statistically significant. For pneumococcus, the VC increased from 25% to 47.7% at the first reassessment and was 33.6% at the second assessment. Thus, there was a significant difference at the first reassessment for pneumococcus, although the study’s findings were negative at 2 years.

Our study has several limitations. First, it only assessed the early impact of the training sessions 8 weeks after the initial assessment at only two of the three centers owing to the COVID-19 pandemic and the national containment procedures. The assessment after the intervention was initially postponed in that center, although it ultimately did not occur to avoid the risk of measurement bias. Delaying the training in the third center could have modified the reassessment of seasonal influenza VC because the influenza epidemic and the national vaccination campaign would have been completed long before the evaluation. There could also have been biases for pneumococcal VC if we reevaluated the effect in the third center at a later time. Indeed, during the containment period, two phenomena were observed. On the one hand, face-to-face consultations were canceled or postponed, the number of telehealth consultations increased, and chemotherapy courses were administered less frequently, which reduced the opportunity for dissemination of vaccination information and the offer to be vaccinated. On the other hand, some physicians assumed that a pneumococcal vaccine could help protect against COVID-19 infection and proposed such vaccinations as a preventative measure [23], [24], [25]. Although our assessment of the effect of the intervention at the third center is incomplete, this remains a multicenter study comprising hospitals with different characteristics.

Second, we considered patients to be vaccinated against pneumococcal infection if they had received an injection of either a 13-valent conjugate vaccine or the full regimen. Data from a later assessment (beyond 8 weeks) could be more clinically relevant. Nevertheless, it seemed relevant to begin by assessing the early effects following the initiation of this regimen by clinicians.

Third, all patients currently receiving systemic cancer treatments at the day hospital (chemotherapy, hormone therapy, immunotherapy, and molecularly targeted therapies) were included. Even if the current recommendations focused on patients exposed to chemotherapy, we decided to include all patients seen at the day hospital for several reasons. Most patients had metastatic cancer and, therefore, had received or will receive chemotherapy. Patients with localized cancer seen at day hospitals currently receive (neo)adjuvant chemotherapy. Patients receiving only hormonal therapies were not included, as they were managed in consultation rather than in the day hospital. Specific recommendations for patients with cancer receiving treatment other than chemotherapy are pending and should be published soon. Some data, however, have already been published; for example, recent studies found that influenza vaccination in patients under tyrosine kinase inhibitors [26] or immunotherapy [27], [28] is safe and effective.

To improve VC, it is necessary to consider everyone’s perceived risk of infection. For example, it may be difficult for physicians or patients to perceive the benefits of pneumococcal vaccination. In fact, the annual incidence of invasive pneumococcal disease ranges from 10 to 100 cases per 100,000 inhabitants [29]. Even with a relative risk up to 23, infection can be considered a rare event.

Other solutions need to be discussed to improve VC in patients with cancer. First, the involvement of general practitioners must improve, as many patients trust their general practitioner, and vaccination training of general practitioners should be improved. The COVID-19 pandemic has shown that many general practitioners favor better collaboration between the city and the hospital. For specialized subjects, optimizing the management of certain pathologies may require better two-way communication. Our training regimen and protocol could help general practitioners improve practices and communication. Second, the establishment of enhanced cooperation between oncologists and infectious disease specialists can increase VC through dedicated consultation or remote expertise. Our study assessed the VC of patients undergoing cancer treatment but not the knowledge of the oncologist or the application of vaccine recommendations. Thus, prescribing a vaccine does not always ensure its administration, and clear and accurate information should be provided at a dedicated time. In medical oncology, finding this time can be difficult. There are three main types of consultations: assessment, day hospital visits, and follow-up consultations. Discussing vaccination during these consultations is complicated; thus, it seems essential to involve another physician in the circuit during in-person or telemedicine consultations dedicated to vaccination discussions. Sitte et al.’s prospective cohort study showed that a specialized infectious disease consultation can improve the VC in patients with gastrointestinal cancer and inflammatory bowel disease [30]. Recently, the implementation of a pre-renal transplant consultation improved VC and patient compliance, with only two refusals of vaccination among 467 patients [31]. Third, our training was intended to help physicians take care of patients. We could have involved other health professionals who work closely with patients, such as nurses. A consultation with a nurse at a day hospital could focus on infectious issues, including fever, febrile neutropenia, catheter-related infection, and vaccinations. Another possibility would be to involve the patient’s relatives to ensure better adherence. Finally, pre-established prescriptions or an immunization page could be included in the personalized patient care plan or inserted at the bottom of letters to the attending physician. In a study by Toleman et al., the intervention consisted of training oncologists and using emails as reminders and for the dissemination of recommendations via intranet and posters in day hospitals. Information was also sent to general practitioners (email) and patients (letters). Involving all health professionals is optimal.

A longitudinal evaluation of VC at later time points after training and studies with larger sample sizes could verify an absence or lack of improvement in VC. It would be interesting to reassess the VC during the next winter season following the implementation of the proposals. Indeed, the COVID-19 pandemic have stressed that vaccine hesitancy and denigration persist, despite the obvious need to protect oneself [32], [33].

Over time, there has been an improvement in the survival and implementation of new therapies, although the number of immunodeficient patients has increased. This is the origin of an increase in the transmission of vaccine-preventable diseases, and everyone must be involved in the fight against these diseases with the help of vaccination.

## Conclusion

Evaluations of the VC of patients with cancer receiving treatment revealed a low VC for DTP, influenza, and pneumococcus during both the first and second evaluation periods. Our intervention did not improve the VC against pneumococcus nor influenza; however, a significant improvement in influenza VC was observed in patients with a poor WHO PS, although there may have been unmeasured cofounders. The findings provide a basis for the concrete implementation of actions to improve VC in the three centers.

## CRediT authorship contribution statement

**Pierre Rivière:** Conceptualization, Resources, Writing – original draft, Writing – review & editing. **Nicolas Penel:** Writing – review & editing. **Karine Faure:** Writing – review & editing. **Guillaume Marie:** Writing – review & editing. **Abeer Najem:** Writing – review & editing. **Marie-Karelle Rivière:** Methodology, Writing – review & editing. **Sophie Panaget:** Conceptualization, Resources, Writing – review & editing.

## Declaration of Competing Interest

The authors declare that they have no known competing financial interests or personal relationships that could have appeared to influence the work reported in this paper.

## Data Availability

Data will be made available on request.
